# Transport Properties of Thermoplastic R-BAPB Polyimide: Molecular Dynamics Simulations and Experiment

**DOI:** 10.3390/polym11111775

**Published:** 2019-10-29

**Authors:** Igor V. Volgin, Maria V. Andreeva, Sergey V. Larin, Andrey L. Didenko, Gleb V. Vaganov, Ilya L. Borisov, Alexey V. Volkov, Leonid I. Klushin, Sergey V. Lyulin

**Affiliations:** 1 Institute of Macromolecular Compounds, Russian Academy of Sciences, Bolshoy pr. V.O., 31, 199004 St. Petersburg, Russia; i.v.volgin@gmail.com (I.V.V.); andreeva.masha@mail.ru (M.V.A.); selarin@macro.ru (S.V.L.); vanilin72@yandex.ru (A.L.D.); glebvaganov@mail.ru (G.V.V.); leo@aub.edu.lb (L.I.K.); 2 A.V. Topchiev Institute of Petrochemical Synthesis, Russian Academy of Sciences, Leninsky pr., 29, 119991 Moscow, Russia; boril@ips.ac.ru (I.L.B.); avolkov@ips.ac.ru (A.V.V.); 3 Department of Physics, American University of Beirut, P.O. Box 11-0236, Beirut 1107 2020, Lebanon

**Keywords:** polyimide, gas separation, polymer membranes, molecular dynamics, simulations

## Abstract

The present work evaluates the transport properties of thermoplastic R-BAPB polyimide based on 1,3-bis(3,3′,4,4′-dicarboxyphenoxy)benzene (dianhydride R) and 4,4′-bis(4-aminophenoxy)biphenyl (diamine BAPB). Both experimental studies and molecular dynamics simulations were applied to estimate the diffusion coefficients and solubilities of various gases, such as helium (He), oxygen (O_2_), nitrogen (N_2_), and methane (CH_4_). The validity of the results obtained was confirmed by studying the correlation of the experimental solubilities and diffusion coefficients of He, O_2_, and N_2_ in R-BAPB, with their critical temperatures and the effective sizes of the gas molecules, respectively. The solubilities obtained in the molecular dynamics simulations are in good quantitative agreement with the experimental data. A good qualitative relationship between the simulation results and the experimental data is also observed when comparing the diffusion coefficients of the gases. Analysis of the Robeson plots shows that R-BAPB has high selectivity for He, N_2_, and CO_2_ separation from CH_4_, which makes it a promising polymer for developing gas-separation membranes. From this point of view, the simulation models developed and validated in the present work may be put to effective use for further investigations into the transport properties of R-BAPB polyimide and nanocomposites based on it.

## 1. Introduction

Separation of gas mixtures is an important requirement in many areas of chemical and petrochemical production. The main tasks of gas separation include the separation of air to produce nitrogen (N_2_) and oxygen (O_2_), the processing of natural gas to produce helium (He), and methane extraction (CH_4_) during the comprehensive preparation of associated petroleum gas [1,2,3]. The efficient performance of these tasks opens up significant opportunities for the reduction of mass greenhouse gas emissions into the atmosphere, the optimization of the processes involved in petrochemical production, and the rational and efficient use of hydrocarbon resources. In recent years, polymer membrane technology was increasingly used [1,2] to address these issues. Currently, polymer membranes are being successfully used for the removal of nitrogen from the air [2], for processing the associated petroleum gas, and for drying natural gas and extracting helium and carbon dioxide from it [3,4].

The low energy consumption of the separation process, along with the portability and relatively low weight of the membrane systems used, are among the main advantages of membrane technology. Another advantage is the scalability of membrane systems, i.e., the possibility of their modular assembly, which increases the efficiency of gas separation [1].

Polymers are perfectly suited for producing gas separation membranes, since their chemical structure can be fine-tuned to obtain materials with the necessary combination of transport and operational characteristics [5,6], together with a required surface geometry and a thin, highly permeable separation layer [7]. The main requirement for polymer membranes is their ability to quickly and efficiently release the desired components of the gas mixture. For this reason, polymers should have high selectivity and permeability values corresponding to the target region of the Robeson plots [8], which depict the existing selectivity–permeability tradeoff for various polymers. Moreover, good resistance to chemically aggressive environments, elevated temperatures, and mechanical stresses are also important for the polymer membranes.

Among the various classes of polymers satisfying these requirements, one can distinguish in particular aromatic polyimides (PI) [6,9]. In recent years, special attention was paid to PIs belonging to a class of polymers with intrinsic microporosity (PIM) [10] or thermally rearrangeable polymers (TRP) [11], whose transport properties approach or even exceed the Robeson upper bound [12]. However, a significant drawback of these PIs is the complexity of their synthesis, and, as a consequence, the high cost of membrane materials based upon them. Current production of polymer gas separation membranes, including composite ones, is based on various commercial PIs, such as Kapton [13], Matrimid 5218 [14,15,16], Upilex-R [13], P84 [17,18], and 6FDA-durene [19], as well as ULTEM [20] polyetherimide, and polybenzimidazole (PBI) [14,16]). For this reason, such polymers were extensively studied by using both experimental and theoretical methods [21,22,23,24]. From a theoretical point, it is worth noting that there are plenty of works by Neyertz et al., who had performed extensive molecular dynamics simulations of various fluorinated 6FDA-based polyimides [25,26,27,28,29,30]. Moreover, Hoffman et al. also examined transport properties of 6FDA-based polyimides [31] and Kapton [32]. A rigorous methodology developed in these works was proven to be an effective tool to address both practical and fundamental issues of polymer gas separation, such as establishing the mechanisms of gas mobility in polymers [26,27,30], calculating gas diffusion and solubility coefficients [26,28,29], and investigating swelling behavior of membranes [25]. Along with these studies, molecular dynamics simulations were also used to reveal correlations between the transport properties of polymers, their chemical structure, and the distribution of free volume in the membranes [33,34,35]. The results of these works showed that, by combining computer simulations and experimental studies, one can construct predictive models which may be used to formulate recommendations for developing new polymer materials with controlled properties.

**Figure 1:**
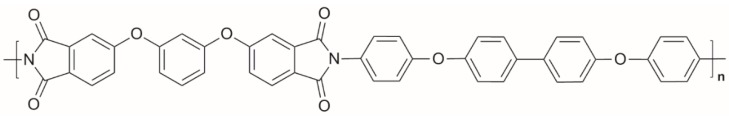
Structure of the R-BAPB elementary unit.

In the present work, we utilize molecular dynamics simulations to investigate the transport properties of the new thermoplastic non-soluble R-BAPB polyimide (Figure 1), which is based on 1,3-bis(3′,4-dicarboxyphenoxy)benzene (dianhydride R) and 4,4′-bis(4″-aminophenoxy)diphenyl (diamine BAPB) [36,37,38,39,40]. Due to its high mechanical strength, melt processability, and crystallization ability, R-BAPB has emerged as a promising polymer binder for producing nanocomposites filled with different types of nanoparticles, as indicated by experimental and computer simulations of its structural and mechanical properties [36,37,38,39,40,41,42,43,44,45,46,47]. Moreover, owing to its non-solubility, R-BAPB can be used to develop highly efficient hollow-fiber membrane materials which will be stable at elevated temperatures and in aggressive media, as required for filtration in the petrochemical industry.

The glass transition temperature of R-BAPB is comparable with that of ULTEM, but lower than those of other commercial polymers (Table 1) used to produce gas separation membranes. On the other hand, R-BAPB’s density is comparable to other polymers, with the exception of Matrimid 5218 and Kapton. The fractional free volume of the R-BAPB exceeds that of Kapton, ULTEM, Upilex-R, and PBI but does not exceed the corresponding values of Matrimid 5218, P84, and 6FDA-durene. Other relevant properties are given in Table S1 (see Supporting Information). All of this, together with the ability of R-BAPB to crystallize in the presence of carbon nanoparticles [36,45], enables us to consider it for the production of polymer membranes being not inferior to those from the widely known commercially available polymers mentioned above.

From this point of view, the lack of information about the transport properties of R-BAPB makes the present work of particular relevance. Previously, we investigated the diffusion of fullerene nanoparticles in R-BAPB polymer melt [48], and here we are applying our extensive expertise to assess for the first time the transport properties of R-BAPB with respect to various gas pairs (He/CH_4_, O_2_/N_2_, CO_2_/CH_4_, He/N_2_, N_2_/CH_4_, CO_2_/N_2_, and He/CO_2_). Moreover, in order to provide comprehensive descriptions, both at macroscopic and microscopic levels, we will supplement our simulations with actual experimental investigations. These important and preliminary investigations aimed at evaluating the transport properties of the polymer itself and developing simulation models will encourage further investigations of nanocomposite membranes based on R-BAPB polyimide, which have a heterogeneous morphology associated with nanoparticle inclusion, and will be performed in the future.

## 2. Materials and Methods

### 2.1. Materials and Methods

Poly(amic acid) (PAA) was synthesized by the polycondensation of 1,3-bis(3,3′,4,4′-dicarboxyphenoxy)benzene (dianhydride R), supplied by “TekhKhimProm” (Yaroslavl, Russia), and 4,4′-bis(4-aminophenoxy)biphenyl (diamine BAPB), supplied by VWR International (Radnor, PA, USA). The purity of the components is more than 97% and 98% for diamine BAPB and dianhydride R, correspondingly. The synthesis was carried out by reacting dianhydride R with diamine BAPB in *N*-methyl-2-pyrrolidone (*N*-MP). During the synthesis, the equimolar ratio of reacting monomers was strictly maintained. The PAA was stirred for 2 h under argon flow. The resulting PAA solution was filtered and degassed under vacuum. The concentration of PAA in the N-MP solution was 20%.

### 2.2. Film Preparation

The R-BAPB film was prepared using a two-stage imidization procedure. Film coatings were formed from the PAA solution. The formation was carried out by pouring a PAA solution onto spatially oriented glass substrates of 2 mm thickness, which were subsequently heated at 353 K for 12 h, to remove the solvent from the bulk. At the second stage, the PAA film coatings were cyclized to obtain PI, after which thermal imidization was performed by stepwise heating for 1 h at 373, 473, and 573 K. The self-supporting films obtained were removed from the substrates and used for further study. The thickness of the resulting sample was 51 ± 2 μm, measured by micrometer.

### 2.3. Characterization of Transport Properties

**Figure 2:**
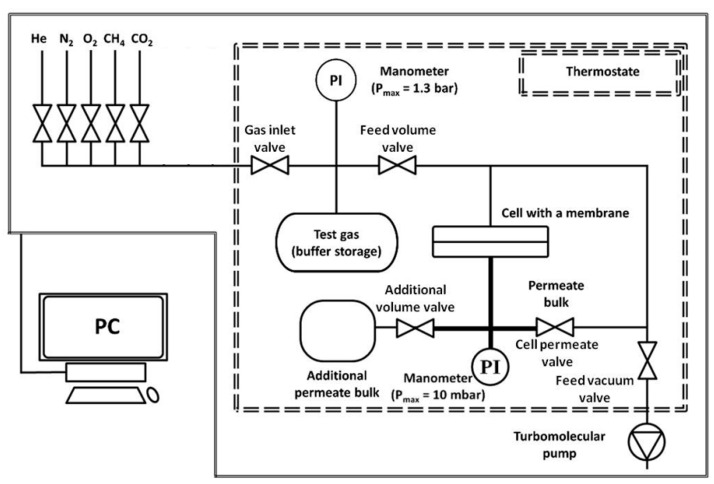
Schematic diagram of the “HZG G&V Permeability Test Unit” apparatus used to determine the gas transport properties of flat membranes.

The experiments on measuring the gas transport properties of the R-BAPB samples were performed on “HZG G&V Permeability Test Unit” (Germany) apparatus. A schematic diagram of the apparatus is shown in Figure 2. The volume of the gas container is 2 liters. The calibrated volume for the permeate is 47.2 cm^3^, and may be increased up to 312.24 cm^3^ to achieve a stable state in the case of high flows or prolonged experiments. Up to eight gas cylinders can be connected to the apparatus simultaneously. The gas pressure above the membrane can be adjusted from 0 to 1.3 bar, while the actual value is detected with a resolution of up to 0.1 mbar. The permeate pressure is measured in the range from 0 to 10 mbar, with a resolution of 0.001 mbar. The diameter of the polymer membrane is 76 mm, but the effective area can be reduced by using appropriate masks. The system is fully automated and computer controlled. The pressure of the gas supply is set by pneumatic valves, and the gases can be alternated automatically.

The key components of the apparatus are located in a thermostatic chamber, which enables measurements to be taken with a preselected temperature program. Supply pressure, permeate pressure, temperature, and automatically calculated permeability are recorded continuously during each measurement cycle. In the present work, we tested membranes with an effective surface area of 13.8 cm^2^ (*d* = 4.19 cm). The measurements were carried out at 303 K, with a partial gas pressure of 0.6 bar.

The chamber with the fixed sample was evacuated for at least 100 h, at a pressure of ~ 10^−7^ bar, using a Pfeiffer Vacuum HiCube 80 Eco turbomolecular pump, before the first measurement, to remove dissolved gases or vapors from the sample and rubber seals. Between the two subsequent gases, the system was also evacuated, purged with a second gas, and again evacuated to ensure the complete removal of the previous gas.

Transport through a polymer film is usually described by a solution–diffusion model [49] that is characterized by three stages: (i) gas absorption at the polymer/gas interface from the supply side, (ii) diffusion of the dissolved gas through the membrane, and (iii) gas desorption from the polymer/gas interface on the low-pressure side.

In simple cases, where the penetrating flow obeys Fick’s law, and the permeate pressure is insignificant compared to the pressure above the membrane, permeability P is usually expressed as the product of the solubility and diffusion coefficients [50]:

(1) P=D·S

where *D* is the diffusion coefficient (cm^2^/s), and *S* is the solubility coefficient (cm^3^(STP)/(cm^3^∙bar)).

**Figure 3:**
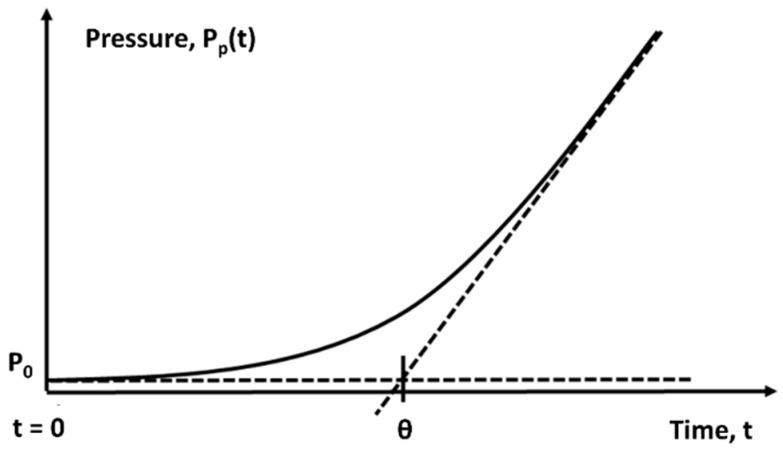
Schematic time dependence of the pressure under the membrane during the experiment.

Determination of gas transport properties is based on measuring the time dependence of the pressure increase in a fixed volume of permeate when a pure gas is exposed to the membrane (see Figure 3).

The permeability coefficient (*P*) is estimated from the slope of the curve in the steady-flux regime, followed by linear extrapolation to zero permeate pressure. The diffusion coefficient is determined by the time delay (*θ*), which can be obtained by linear extrapolation of the steady-flux pressure curve to the initial pressure (*P_0_*) [49]:

(2) $$D=\frac{l^{2}}{6\theta },$$

where *l* is the membrane thickness.

The speed of the sensors is less than 0.05 s; therefore, the delay time (*θ*), up to 0.5 s, can be determined with an error of less than 10%.

Subsequently, the solubility value can be obtained from the estimated permeability coefficient within the following equation:

(3) $$S=\frac{P}{D}.$$

Finally, the separation performance of the membrane material is characterized by an ideal selectivity (*α*), defined as the ratio of the permeability coefficients of pure gases *A* and *B*:

(4) $$\alpha_{A/B}=P_{A}/P_{B}.$$

### 2.4. Molecular Dynamics Simulations

#### 2.4.1. Simulation Details

An analysis of the works, considering molecular dynamics simulations of polymer transport properties [25,26,27,28,29,30,31,32,33,34,35] indicates that the interactions of gas molecules with each other and with polymers may be described using two approaches. In most cases, parametrization of the interactions is based on the use of atomic types and the corresponding parameters already available in the force field [31,32,33,34,35]; however, some authors implement additional interaction parameters to the original force field, parameters which were developed specifically to describe the interactions between gas molecules [25,26,27,28,29,30].

Both of these approaches enable good qualitative conclusions to be drawn about the transport properties of polymers, in accordance with experimental data, despite the lack of exact quantitative correlations. Moreover, these approaches enable the accurate reproduction of the fundamental dependences between diffusion coefficients, activation energies, or solubilities of gases and the effective diameters of their molecules [51,52].

In the present work, interactions between the atoms of R-BAPB are described within the framework of the Gromos53a6 force field [53,54]. This force field belongs to the open-source force fields of the class I and is native for Gromacs [55] software package, which is perfectly suited for performing computationally demanding microsecond timescale all-atom molecular dynamics simulations. As it was shown previously, Gromos53a6 may be successfully used to simulate thermophysical, mechanical, and relaxation properties of various thermoplastic polyimides, and even the initial stages of their crystallization in the presence of the carbon nanoparticles [43,44,45,46,56,57,58,59].

However, one of the main problems of Gromos53a6 force field is that it has no special atomic types for the gas molecules (He, N_2_, O_2_, and CH_4_). Therefore, in accordance with common practice, we have used additional parameters, which are calibrated for the thermodynamic, structural, and dynamic properties of the gases in question [28,60,61,62,63]. The parameters for He were proposed by Lee et al. [28,60]; for N_2_ by Fischer and Lago [61]; for O_2_ by Cheung and Powles [62]; and for CH_4_ by Yin and MacKerell [63], and are summarized in Table 2. Finally, non-bonded parameters for “gas-polymer” interactions were calculated using standard geometric combination rules implemented in the Gromos53a6 force field [53,54]. We show that implementation of the parameters for gas molecules into Gromos53a6 allows us to reproduce quantitative trends and correlations between solubility and diffusivity of the gases observed in the experiment.

Another important issue is related to accounting for Coulomb interactions in the system due to the presence of partial charges on the atoms of both the gases and the polymer [44,64]. As previously demonstrated, Coulomb interactions can lead to a sharp decrease in both the translational and segmental mobility of polymer chains, even at high temperatures (~600 K) [56,57,58,59]. It suggests that longer simulations are needed to determine the diffusion coefficients of gases in polymer, especially at room temperature, something which is currently beyond the power of modern supercomputers. Therefore, from the point of view of determining the diffusion properties of gas molecules, the absence of partial charges on polymer atoms can significantly facilitate the calculation of the polymers’ transport properties. On the other hand, it is worth noting that the gases examined in the present work do not have dipole moments, nor does R-BAPB contain highly polar groups in its structure. Thus, in a first approximation, we can assume partial charges to be equal to zero, due to the small contribution of dipole–dipole interactions to the transport properties of R-BAPB.

Equilibrium configurations of R-BAPB, having 27 polymer chains with a degree of polymerization *n* = 8 (the total number of atoms in the system is 17766), were taken from [45]. For details of the equilibration procedure, we refer the reader to our previous work [65]. The edge of the simulation cubic cell is ~6 nm, with periodic boundary conditions imposed in all directions. Computer simulations were performed using the Gromacs software package [55] in an NpT ensemble at a pressure of 1 bar at different temperatures (430, 440, 450, and 470 K). A Nosé–Hoover thermostat [66,67] and a Parrinello–Rahman barostat [68,69] were used to maintain temperature and pressure values at the required level. Time constants for the thermostat and barostat were *τ_T_* = 0.5 ps and *τ_p_* = 2.5 ps, respectively. Equations of motion were integrated every 2 fs for temperatures *T* < 430 K and 1 fs for temperatures *T* > 430 K. The cut-off radius of the non-bonded interactions was taken to be 1 nm.

The initial configurations necessary for performing calculations of diffusion and solubility coefficients were produced by cooling the equilibrium configurations of the R-BAPB from 600 to 300 K. The systems were cooled stepwise, i.e., the temperature was decreased instantly by 10 K every 4 ns, corresponding to an effective cooling rate of 1.5 ∗ 10^11^ K/min. This technique is widely used to obtain systems in molecular dynamics simulations to study the thermophysical and mechanical properties of polymers [70], including thermoplastic polyimides [44,46,57,59].

The resulting R-BAPB configurations were used to create systems containing gas molecules. The insertion of gas molecules into the polymer was performed using the *gmx insert-molecules* utility, implemented in the Gromacs software package. The gas molecules insertion was followed by a two-stage equilibration. Initially, the steepest descent algorithm was used to minimize the potential energy of the systems, after which a short simulation run in the *NVT* ensemble was performed for 1 ns, which allowed the energy of the systems to relax properly (the corresponding dependencies are presented in Supporting Information, Figure S1).

#### 2.4.2. Solubility Calculations

The gas solubility in R-BAPB was estimated using the test particle insertion method proposed by Widom [71]. This method is based on the random insertion of a test particle into a simulation cell with the polymer and subsequent calculation of the change to the system’s potential energy (*∆U*), corresponding to the insertion of the test particle. The excess chemical potential (*μ_ex_*) of the test particle insertion into the system is calculated from the *∆U*, according to the following formula:

(5) $$\mu_{\mathrm{ex}}=-k_{B}T\cdot \ln\langle \exp\left(-\frac{\Delta U}{k_{B}T}\right)\rangle ,$$

where angle brackets indicate averaging over the number of test insertions.

The excess chemical potential calculated at a given temperature is then used to estimate the gas solubility coefficient, which is typically brought to standard temperature and pressure (STP) conditions of *T* = 273.15 K and *P* = 1 bar [52]:

(6) $$S=V_{\mathrm{mol}}\cdot \frac{1}{RT}\cdot \exp\left(-\frac{\mu_{\mathrm{ex}}}{RT}\right),$$

where *R* is the gas constant, and *V_mol_* is the molar volume of the gas at STP. Thus, the solubility unit is $\frac{cm^{3}\left(STP\right)}{cm^{3}*bar}$.

Five trajectories of R-BAPB at 300 K were used for the solubility calculations. The recording time step was 5 fs for each trajectory. The trajectories were simulated from the configurations obtained after cooling R-BAPB from 600 K, according to the method described above. It is worth noting that these trajectories may be considered to be independent, since the time window between configurations taken for the cooling procedure is 100 ns, which is an order of magnitude longer than the density relaxation times at 600 K [43]. The number of test insertions per frame was 10^5^. Further increase in the number of test insertions does not lead to a significant change in the solubility values within the error, as the additional analysis shows (see Supporting Information, Figure S2), while fewer insertions lead to an overestimation of the solubility coefficients.

The solubility value obtained was used to calculate the equilibrium value of the number of gas molecules *n* (i.e., the concentration at a fixed volume of the simulation cell) in the polymer, according to the following formula [24]:

(7) $$n\approx \frac{P*V_{p}}{k_{B}T}\cdot \exp\left(-\frac{\mu_{\mathrm{ex}}}{k_{B}T}\right),$$

where *V_p_* is the volume of the simulation cell, and *P* is the pressure.

#### 2.4.3. Diffusion Coefficient Calculations

Determination of the transport properties of polymers is associated with the calculation of gas diffusion coefficients at room temperature. In computer modelling, one of the main characteristics which provides information on the dynamic properties of gas molecules in a polymer is the mean squared displacement function (MSD), which shows how far the center of mass of the gas molecule is displaced over time (*t*):

(8) $$\Delta r_{\mathrm{com}}^{2}\left(\Delta t\right)=\langle {\left|r\left(\Delta t\right)-r\left(0\right)\right|}^{2}\rangle ,$$

where the angle brackets denote averaging over time and the number of gas molecules in the system.

In general, the time dependence of the MSD conforms to a power law curve: $\Delta r_{com}^{2}\left(\Delta t\right)\sim \Delta t^{a}$. Here, three different diffusion regimes can be distinguished: a ballistic regime in small time scales (*a* = 2), a subdiffusive regime (0 < *a* < 1), and the normal diffusion regime (*a* = 1) [72]. In this particular case, determination of the gas diffusion coefficient in the polymer must be carried out on the basis of analysis of the MSD in the normal diffusion regime, when the diffusion of the particles is through random walks [72]. In this instance, in accordance with Einstein’s equation, within large time limits, the MSD function is proportional to time, and the proportionality coefficient defines the diffusion coefficient (*D*):

(9) $$\Delta r_{\mathrm{com}}^{2}\left(\Delta t\right)=6D\Delta t.$$

However, in glassy polymers, the mobility of polymer chains is significantly reduced compared to their mobility in a molten state, and, as a result, it can often take a considerable time (of the order of several microseconds) to establish a normal diffusion regime of the gas molecules. Such a long simulation of systems consisting of tens of thousands of atoms is a very demanding task, often beyond the capability of the resources available, making it impossible to directly determine the diffusion coefficient at room temperature. A solution to this problem can be found via the use of various indirect methods for diffusion coefficient estimation [73,74]. In this work, we have used a method based on the construction of the Arrhenius equation, which plots the dependence of the gas diffusion coefficient on the reciprocal temperature at which the measurement takes place. To obtain this dependence, the diffusion coefficients of the gases were calculated at temperatures lower than the glass transition temperature of the polymer. In this case, according to the activation approach to the description of gas diffusion in polymers, the temperature dependence of the diffusion coefficient obeys the Arrhenius Law [52]:

(10) $$D=D_{0}\cdot \exp\left(-\frac{E_{D}}{RT}\right),$$

where *D_0_* is the pre-exponential factor, and *E_D_* is the diffusion activation energy.

On a semi-logarithmic scale, the Arrhenius plot (i.e., the logarithm of the diffusion coefficient versus the reciprocal temperature) is a linear function. Arrhenius plotting is often used to determine diffusion activation energy [75,76,77], and extrapolating this dependence to the low temperature region allows one to determine the diffusion coefficients of gases [25] in the temperature range of interest.

## 3. Results and Discussion

In this section, we present the results of measuring the transport properties of R-BAPB in the experiment and molecular dynamics simulations.

### 3.1. Solubility

**Figure 4:**
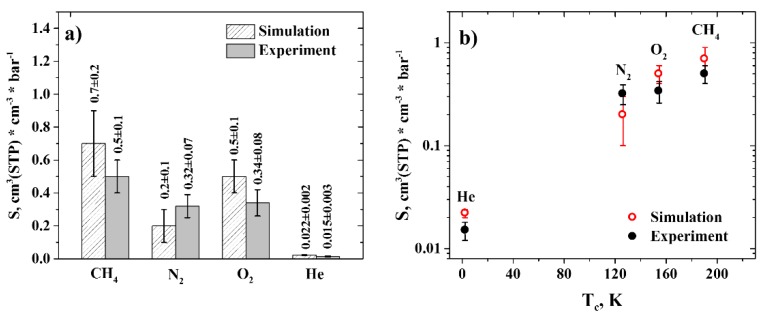
(**a**) Solubilities of gases in R-BAPB under STP conditions obtained in molecular dynamics simulations (shaded columns) and in experiment (solid columns). (**b**) Solubilities of gases in R-BAPB versus their critical temperatures obtained in molecular dynamics simulations (blank red circles) and in experiment (solid black circles). Semi-logarithmic axes. The error bars denote standard deviation.

Gas solubility coefficients in molecular dynamics simulations were estimated from the excess chemical potentials of gas molecules insertions in R-BAPB, calculated at *T* = 300 K. On the other hand, experimental solubility coefficients were estimated from the diffusivity and permeability data obtained at 303 K. Both simulational and experimental solubility coefficients were brought to the STP conditions, in order to compare them correctly. The results are presented in Figure 4a.

As can be seen from Figure 4a, the difference between the average values of the solubility coefficients is 40% (for CH_4_), 37.5% (for N_2_), 47% (for O_2_), and 47% (for He). However, we could conclude about the good agreement between the results, since the average values of the solubility coefficients are close to each other, taking into account the error bars. This agreement proves the adequacy of the parameters describing interactions between the gas molecules and the polymer. Moreover, the solubilities of the gases display a linear dependence on the gases’ respective critical temperatures, Figure 4b, in accordance with the literature data [52], which further indicates the reliability of the results obtained.

### 3.2. Diffusion Coefficients

Simulating gas diffusion requires an accurate estimation of the number of gas molecules to be considered in the simulation cell. As was shown earlier, the number of gas molecules absorbed in the polymer membrane at a given temperature and pressure can be calculated from the excess chemical potential of the gases (according to Equation (7)). These calculations lead to the results shown in Table 3.

As can be seen, the limiting number of gas molecules in the simulation cell under equilibrium sorption and STP conditions varies from 0.1 (He) to 4 (CH_4_) molecules. In the case of He, this is due to its low solubility in R-BAPB, which means that He molecules are practically absent in the simulation cell in question. Given such small values for the equilibrium concentration of the gases, as well as the fact that gas solubility decreases with increasing temperature, the question arises about the correct choice of the number of particles for studying the gas molecules’ mobility, especially at elevated temperatures.

On the one hand, choosing a small number of molecules may lead to results having insufficient statistical significance, while increasing the number of independent samples in this case will lead to a considerable increase in the computational cost of simulations. On the other hand, if too many gas molecules are present in the system, a polymer swelling may occur [25,27], which has an undesirable effect, since, in this case, the diffusion properties of the gas molecules are altered.

An efficient way to increase the concentration of gas molecules while maintaining the correlation between concentration and solubility is to apply an external pressure or to increase the size of the simulation cell [24]. However, in the first instance, this may make it necessary to apply different pressures while studying different gases, which is undesirable for comparison of the simulation results, as well as possibly leading to structural changes in the polymer. In the second case, increasing the system size will lead to a sharp increase in the computational cost of simulations, as in the case of a larger number of independent samples.

Therefore, in order to select the optimal number of gas molecules, we performed preliminary simulations of gas diffusion in systems containing a different number of gas molecules (namely, He or CH_4_) at 470 K. An analysis of the MSD curves of the gases (given in Supporting Information, Figure S4) showed that gas dynamics tend to increase upon increasing the concentration of the gas molecules, especially when the number of molecules in the system is above 20. This may be a consequence of polymer swelling; thus, only 10 gas molecules were used to determine their diffusion coefficients in R-BAPB, in order to negate the possible influence of polymer swelling.

**Figure 5:**
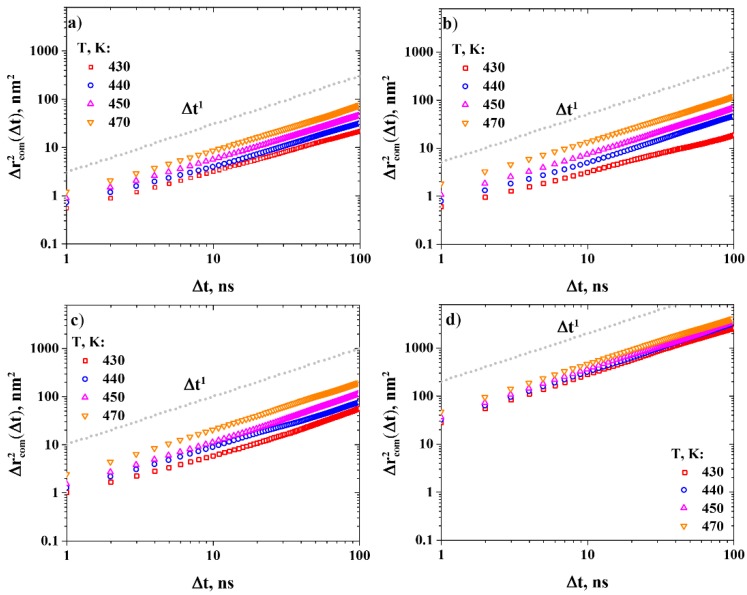
Time dependence of the mean squared displacement (MSD) of (**a**) CH_4_, (**b**) N_2_, (**c**) O_2_, and (**d**) He in R-BAPB, at various temperatures. Double-logarithmic axes.

Having established the optimal concentration of the gas molecules in the R-BAPB samples, the diffusion coefficients of the gas molecules were estimated. To this end, we performed simulations of gas diffusion in a wide temperature range below the glass transition temperature of R-BAPB (*T_g_* = 485 K) on timescales from 500 to 1000 ns, depending on the temperature considered. Then, the MSD functions of the gas molecules were calculated (see Figure 5).

**Figure 6:**
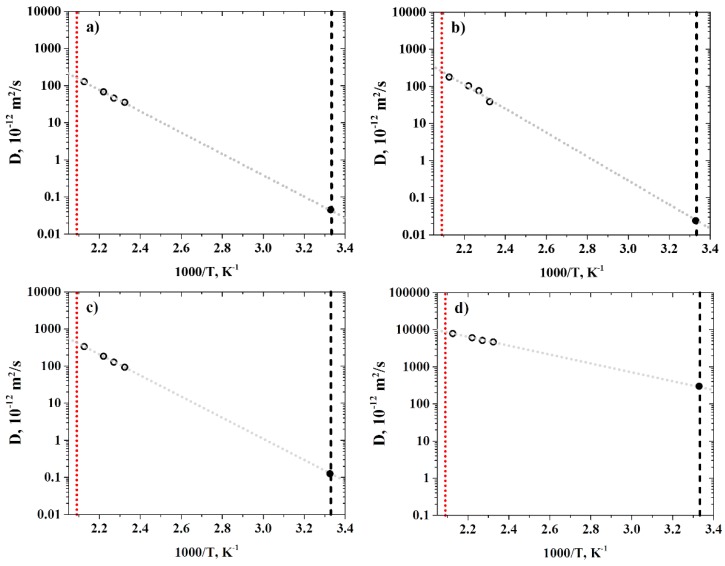
Dependence of the diffusion coefficients of (**a**) CH_4_, (**b**) N_2_, (**c**) O_2_, and (**d**) He in R-BAPB on the reciprocal temperature obtained in molecular dynamics simulations. Semi-logarithmic axes. The red dotted line indicates the glass transition temperature of R-BAPB. The black dashed line corresponds to *T* = 300 K. The solid black symbols indicate the diffusion coefficients at *T* = 300 K, obtained by extrapolating the diffusion coefficients at higher temperatures, using the Arrhenius Law. The error bars are of the size of the symbols.

The diffusion coefficients of the gases were calculated from the MSD curves (Figure 6) corresponding to the timescales of the normal diffusion regime (the values of MSD slopes are presented in Supporting Information, Figure S4).

The errors in the diffusion coefficients of gases at high temperatures (430, 440, 450, and 470 K) were determined as a standard deviation in the diffusion coefficients calculated from different sections of the MSD curves in the normal diffusion regime. At the same time, the relative error of the experimental data is ~23%. The diffusion coefficients of the gases in R-BAPB at *T* = 300 K were derived by extrapolating the diffusion coefficients at higher temperatures, using the Arrhenius Law. In this case, the error of the diffusion coefficients at room temperature was determined as the error of the corresponding approximation.

**Figure 7:**
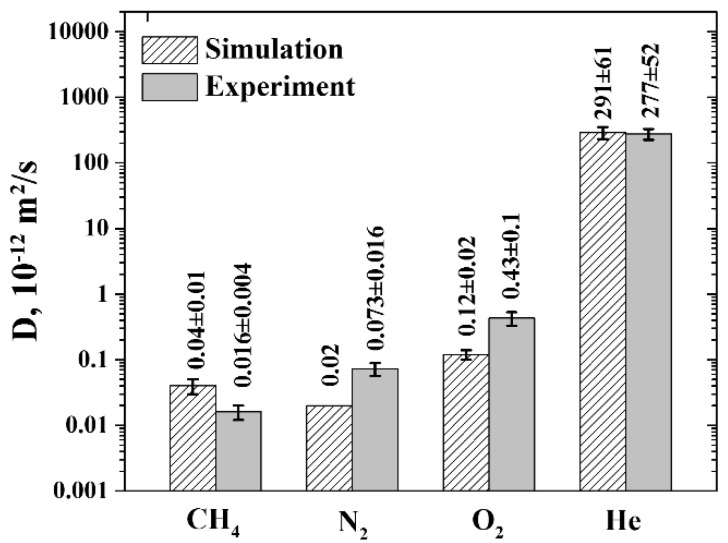
Diffusion coefficients of gases in R-BAPB at temperature *T* = 300 K, obtained in molecular dynamics simulations (shaded columns) and in the experiment (solid columns). Semi-logarithmic axes. The error bars denote standard deviation (see main text for details). The diffusion coefficient of N_2_ lies in the range (0.007 ÷ 0.09) × 10^−3^ nm^2^/ns, with 0.02 nm^2^/ns being the value given by the approximation line.

The diffusion coefficients of the gases obtained from the experiment and molecular dynamics simulations are given in Figure 7 for comparison.

As can be seen from Figure 7, the diffusion coefficients of CH_4_ is approximately 2–3 times larger than the corresponding experimental values, while the diffusion coefficients of O_2_ and N_2_, on the contrary, are approximately 2–3 times lower. Only for He the desirable consistency is observed. Therefore, in spite of the observed qualitative and quantitative agreement between the solubilities of the gases in the experiment and simulations, the situation with the diffusion coefficients turns out to be more complicated. Nevertheless, a quantitative conclusion could still be drawn about the transport properties of R-BAPB with respect to He, O_2_, and N_2_, since the experimentally relationship between their diffusion coefficients (*D_He_* > *D_O2_* > *D_N2_*) is preserved in the simulations.

**Figure 8:**
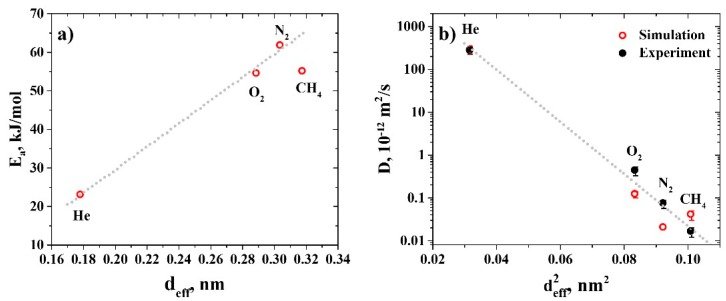
(**a**) Dependence of the activation energy of diffusion of the gases in R-BAPB on their effective diameter [51], obtained in molecular dynamics simulations. (**b**) The values of the diffusion coefficients of gases in R-BAPB at *T* = 300 K at their effective diameters [51], obtained in molecular dynamics simulations (blank red circles) and the experiment (solid black circles). Semi-logarithmic axes. The error bars denote the standard deviation (see main text for details). The gray lines are to guide the eye.

The reason for the diffusion coefficient of CH_4_ being slightly larger than that of N_2_ could be discerned by analyzing the dependence of the activation energies of gas diffusion on the effective diameter of the gas molecules (see Figure 8a). As can be seen, for He, N_2_, and O_2_, diffusion activation energies depend linearly on the effective diameter of the gases involved, as is typically observed in the literature [52]. However, the activation energy for CH_4_ lies below the linear dependence. The same deviation is observed when considering the dependence of the diffusion coefficients of gases on the squared effective diameter of the gas molecules (see Figure 8b). A possible reason for this deviation may be related to the absence of partial charges on CH_4_ atoms in the simulations. Verification of this assumption will be the subject of our further investigations.

### 3.3. Permeability and Selectivity

**Figure 9:**
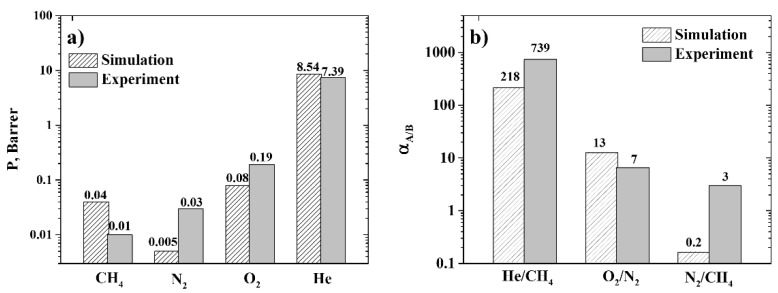
(**a**) Permeability of R-BAPB with respect to CH_4_, N_2_, O_2_, and He at *T* = 300 K. (**b**) Selectivity of R-BAPB for various gas pairs at *T* = 300 K, obtained in molecular dynamics simulations and in experiment. The results obtained in molecular dynamics simulations are shown by the shaded columns, and experimental data are given in the solid columns. Semi-logarithmic axes.

Given the values of the diffusion and solubility coefficients of the gases, we have calculated the permeability and selectivity of R-BAPB (see Figure 9).

As can be seen from Figure 9a, molecular dynamics simulations enable the reproduction of the experimentally observed difference in the permeability of R-BAPB with respect to different gases, at least in qualitative terms. An exception is CH_4_, for which the R-BAPB permeability is significantly lower than the corresponding experimental value, which in turn is due to an underestimation of the diffusion coefficient in the simulations discussed above.

Nevertheless, analysis of the simulation results enables us to draw a qualitative conclusion about the prospects for using R-BAPB for He extraction, due to its high He/CH_4_ selectivity (around 700), which is also confirmed in the experiment (see Figure 9b). From Figure 9b, it can also be seen that simulations are capable of reproducing the qualitative difference in the R-BAPB selectivities observed in the experiment. At the same time, the quantitative difference between the selectivity values in the experiment and simulations owes more to the kinetic component of the selectivity than its thermodynamic aspect, which could be attributed to the difference in the structure of the free volume of the R-BAPB samples in molecular dynamics simulations and the experiment, and will be investigated further.

**Figure 10:**
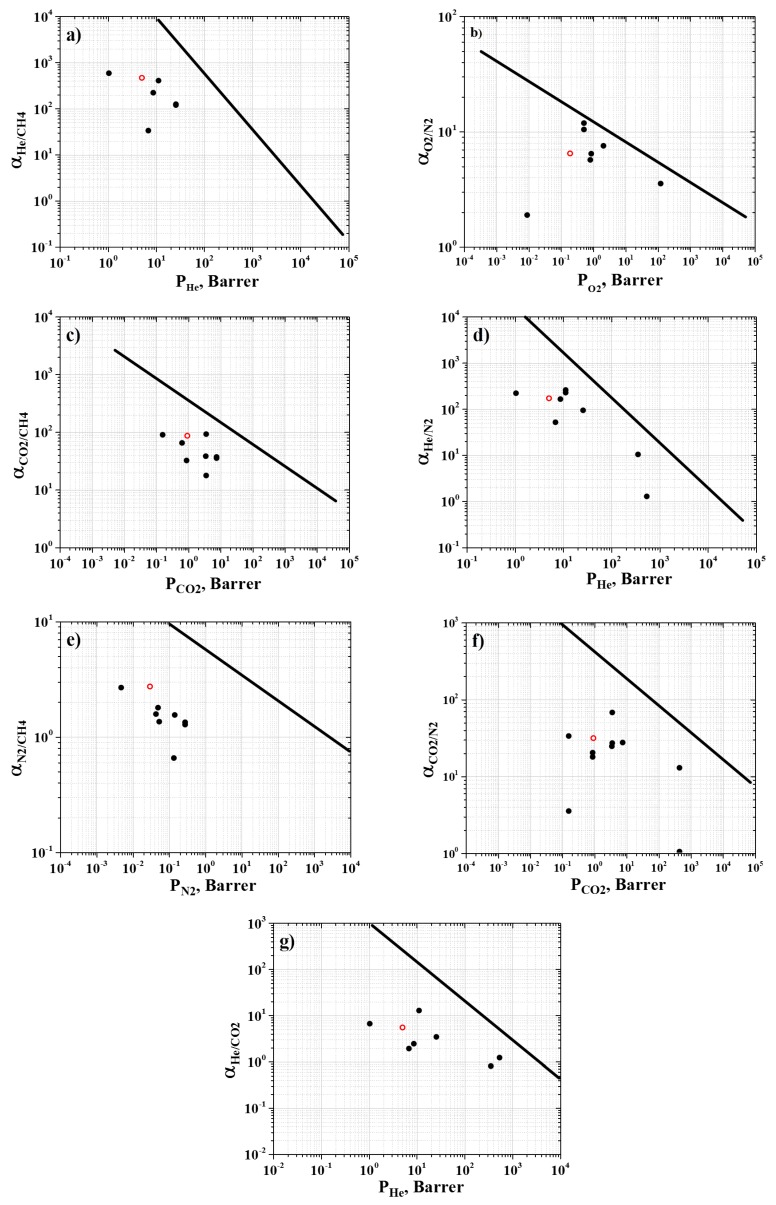
Robeson plots for (**a**) He/CH_4_, (**b**) O_2_/N_2_, (**c**) CO_2_/CH_4_, (**d**) He/N_2_, (**e**) N_2_/CH_4_, (**f**) CO_2_/N_2_, and (**g**) He/CO_2_. Solid black dots indicate data for commercial PIs available in the literature [13,14,15,16,17,18,19,20]. Open red symbols correspond to the experimental results on the transport properties of R-BAPB obtained in the present work. Solid lines indicate the Robeson upper bound [8].

Finally, the results of the experimental investigation into R-BAPB’s transport properties were plotted on the Robeson plots [8] (see Figure 10). 

Analysis of the Robeson plots (see Figure 10) shows that all the points corresponding to the polymers, including R-BAPB, lie below the Robeson upper bound. Nevertheless, R-BAPB has rather good selectivities with respect to He, N_2_, and CO_2_, (see Figure 10a,c,e), since they are comparable to the values for other commercial PIs (such as Kapton, ULTEM, Upilex-R, Matrimid 5218, P84, and 6FDA-durene) [13,14,15,16,17,18,19,20], which are typically used to develop efficient gas separation membranes [6]. In the next stage of our research, we will evaluate transport properties of R-BAPB with respect to gas mixtures.

In summary, the results of the comparison of R-BAPB separation performance with other commercial PIs allow us to consider R-BAPB in further investigations. Particularly, we think that the addition of carbon nanoparticles may be an efficient way to improve its performance. It would be important to assess transport properties of membranes based on R-BAPB polyimide filled with carbon nanotubes, since their addition may be used to control microstructure due to the polyimide crystallization in the nanocomposite, as it was shown earlier, in our previous works [36,45]. From this point of view, the reliable computational models developed and validated in the present work may provide valuable insights into the transport properties of such mixed-matrix membranes and into the role of the interphase region between polymer and nanoparticle in its performance.

## 4. Conclusions

In the present work, the transport properties of the promising thermoplastic R-BAPB polyimide were evaluated for the first time, using comprehensive experimental and molecular dynamics simulations studies.

The solubility and diffusion coefficients of various gases, such as He, O_2_, N_2_, and CH_4_, were estimated. The simulation results on solubility coefficients of gases were found to be in good quantitative and qualitative agreement with the corresponding experimental data. Computer simulations also reproduced qualitatively the relationship between the diffusion coefficient values for He, O_2_, and N_2_ observed in the experiment. The reliability of the results was also confirmed while examining the dependencies of the activation energies and diffusion coefficients of the gases on the effective diameters of the gas molecules. Nevertheless, the quantitative difference between the simulation and experimental results calls for further investigations aimed at elucidating possible routes for its improvement by simultaneous experimental and simulation studies.

Finally, by placing the experimental data on the Robeson plots, we found that R-BAPB selectivity performance is fairly well comparable to those of other commercial polymers, especially for the separation of N_2_, CO_2_, and He from CH_4_. This should stimulate further optimization of polymer gas separation membranes based on R-BAPB polyimide and the development of mixed-matrix membranes based on it, which may be potentially competitive to other commercial membranes.

## Figures and Tables

**Table 1:** Properties of R-BAPB and commercially available polyimides used in membrane gas separation.

	R-BAPB	Kapton [13]	ULTEM [20]	Upilex-R [13]	Matrimid 5218 [15]	P84 [18]	6FDA-Durene [19]	PBI [16]
T_g_, K	485	693	488	543	583	573	697	690
ρ, kg/m^3^	1324	1395	1280–1300	1366	1.172	1336	1333	1311
FFV,%	13.9	12.9	10.8–11.5	12.1	26.8	20.3	18.0	11.6

**Table 2:** Parameters for van der Waals and electrostatic interactions of atoms of the studied gases.

Gas	Atom	Interaction Potential Parameters		Reference
		ε/k_B_, K	σ, Å	
He	He	6.030	2.6282	[28,60]
N_2_	N	37.296	3.31	[61]
O_2_	O	44.6	3.09	[62]
CH_4_	C	47.8	3.7595	[63]
	H	8.5546	2.3876	

**Table 3:** The number of gas molecules in the simulation cell at *T* = 300 K, calculated on the basis of the excess chemical potentials obtained in molecular dynamics simulations.

Gas	Number of Gas Molecules
CH_4_	4
N_2_	1
O_2_	3
He	0.1

## Supplementary Materials

The following are available online at https://www.mdpi.com/2073-4360/11/11/1775/s1. Table S1: Properties of R-BAPB thin film membrane considered in the work. Figure S1: Time dependence of the potential, kinetic, and total energies of R-BAPB sample containing CH_4_, O_2_, N_2_, or He at different temperatures after energy minimization. Figure S2: Dependence of the solubility coefficient of CH_4_, O_2_, N_2_, or He at STP conditions calculated from a single trajectory obtained at T = 300 K on the number of the test particle insertions. Figure S3: Time dependence of mean squared displacement of He or CH_4_ gas molecules in R-BAPB at different concentrations (number of gas molecules) at T = 470 K, calculated over 100 ns. Figure S4: Time dependence of the slope of the mean squared displacement of CH_4_, O_2_, N_2_, or He molecules in R-BAPB at different temperatures.
