# Supplementary material for: Transport Properties of Thermoplastic R-BAPB Polyimide: Molecular Dynamics Simulations and Experiment
Source: Polymers (Basel). 2019 Oct 29;11(11):1775. doi: 10.3390/polym11111775 (PMC6918166; doi:10.3390/polym11111775)
Supplement: Supplementary file 1 [file polymers-11-01775-s001.pdf]

Electronic Supplementary Information

to

Transport properties of thermoplastic R-BAPB polyimide: molecular-dynamics simulations and experiment

I.V. Volgin[1], M.V. Andreeva[1], S.V. Larin[1], A.L. Didenko[1], G.V. Vaganov[1], I.L. Borisov[2], A.V. Volkov[2], L.I. Klushin[1,3], S.V. Lyulin[1,*]

[1] *Institute of Macromolecular Compounds, Russian Academy of Sciences, Russian Federation, St. Petersburg, 199004, Bolshoy pr. V.O., 31.*

[2] *A.V. Topchiev Institute of Petrochemical Synthesis, Russian Academy of Sciences, Russian Federation, Moscow, 119991, Leninsky pr., 29.*

[3] *American University of Beirut, Department of Physics, Lebanon, Beirut 1107 2020, P.O. Box 11-0236.*

***Corresponding author, e-mail: Sergey V. Lyulin, s.v.lyulin@gmail.com**

Table S1. Properties of R-BAPB thin film membrane considered in the work.

T_g, K	T_m, K	τ_5, K	σ, MPa	E, MPa	ε, %
485	599	805	108.4±13.5	2514±104	95.2±33.2
T_g – glass transition temperature, T_m – melting temperature, τ_5 – decomposition temperature, σ – tensile strength, E – elasticity modulus, ε – tensile strain.					

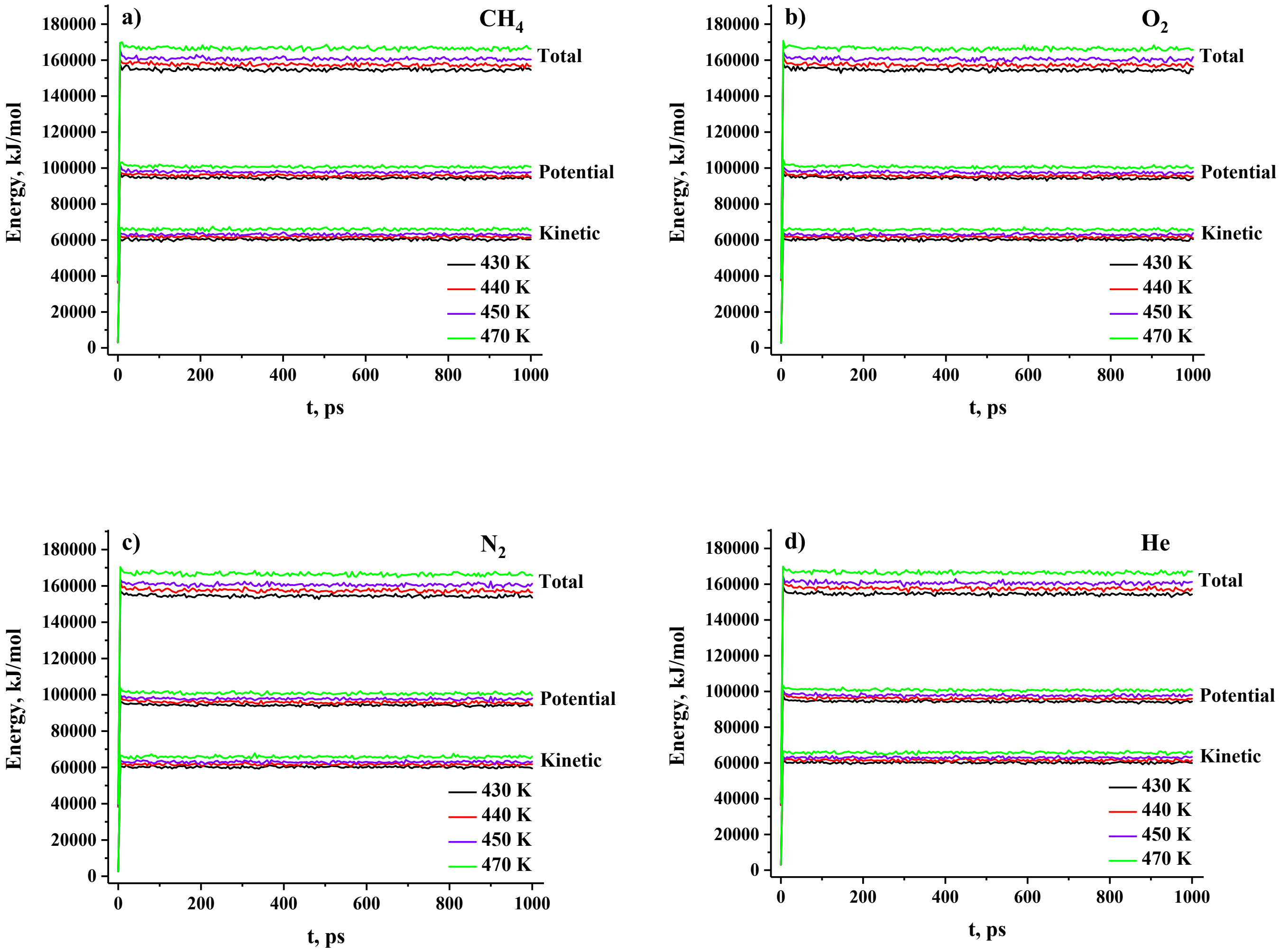

Figure S1. Time dependence of the potential, kinetic and total energies of R-BAPB sample containing a) CH_4, b) O_2, c) N_2, or d) He at different temperatures after energy minimization.

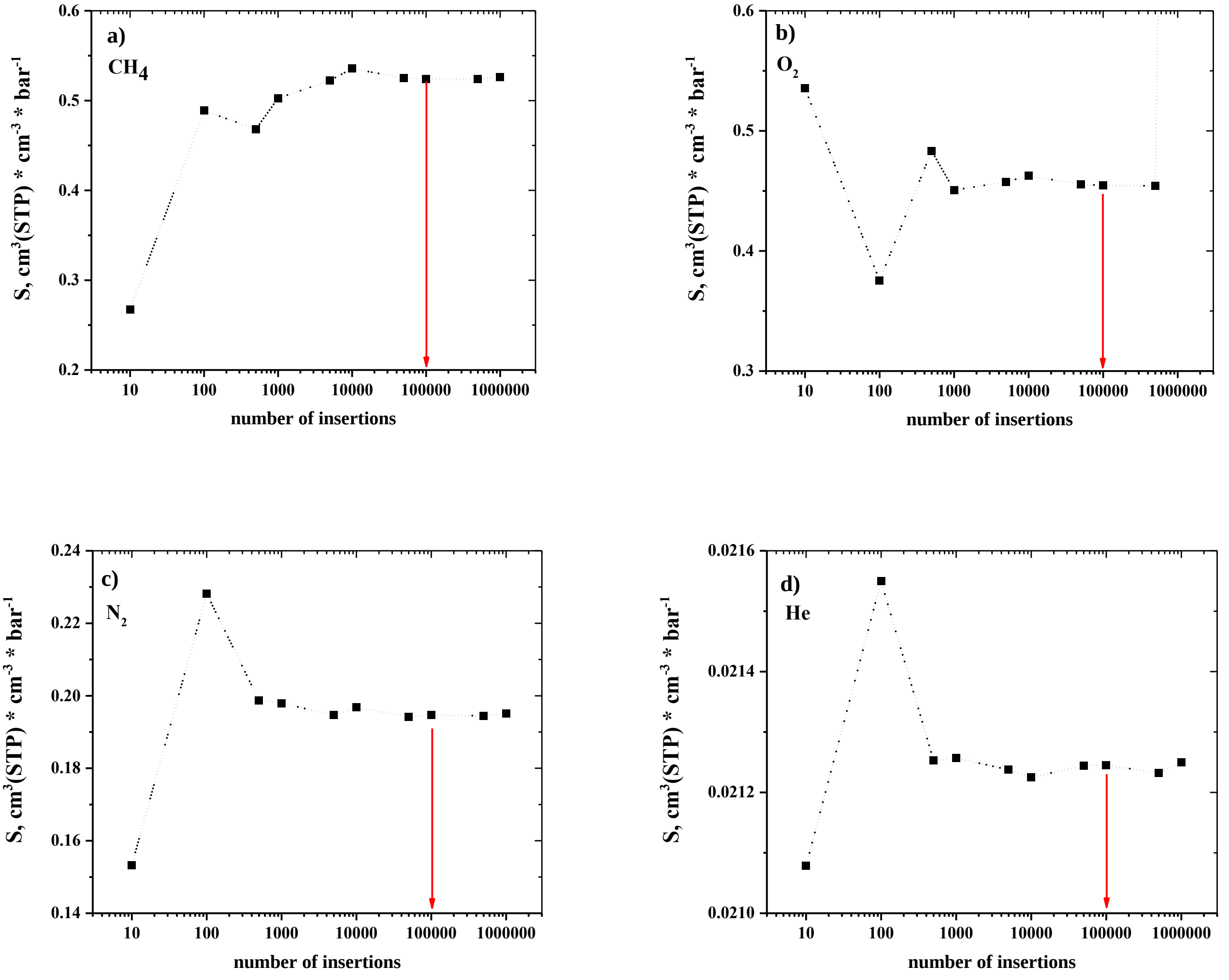

Figure S2. Dependence of the solubility coefficient of a) CH_4, b) O_2, c) N_2, or d) He at STP conditions calculated from a single trajectory obtained at T = 300 K on the number of the test particle insertions. The arrow denotes an optimal number of insertions (n = 100000).

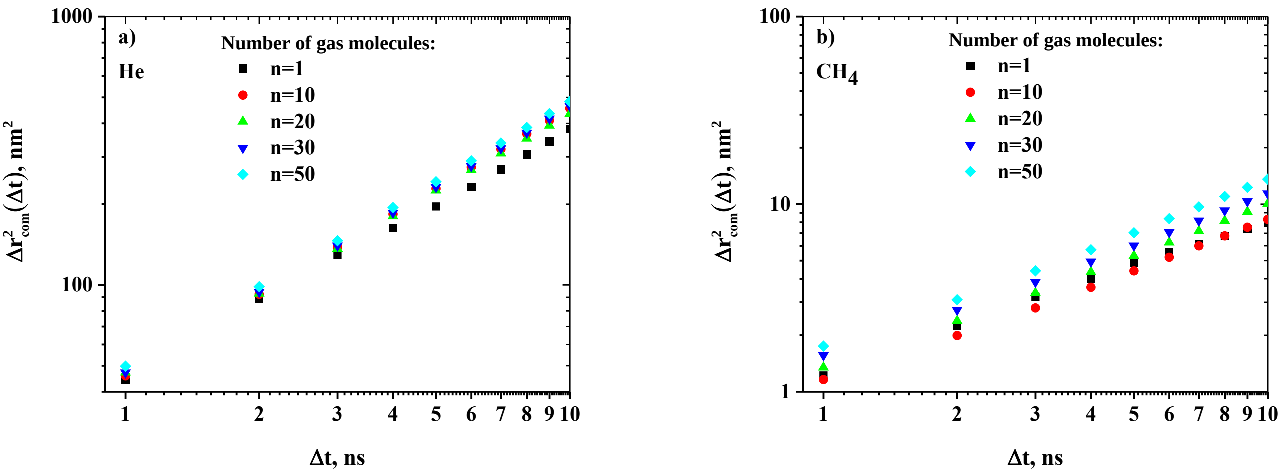

Figure S3. Time-dependence of mean squared displacement of a) He or b) CH_4 gas molecules in R-BAPB at different concentrations (number of gas molecules in the system) at T = 470 K calculated over 100 ns.

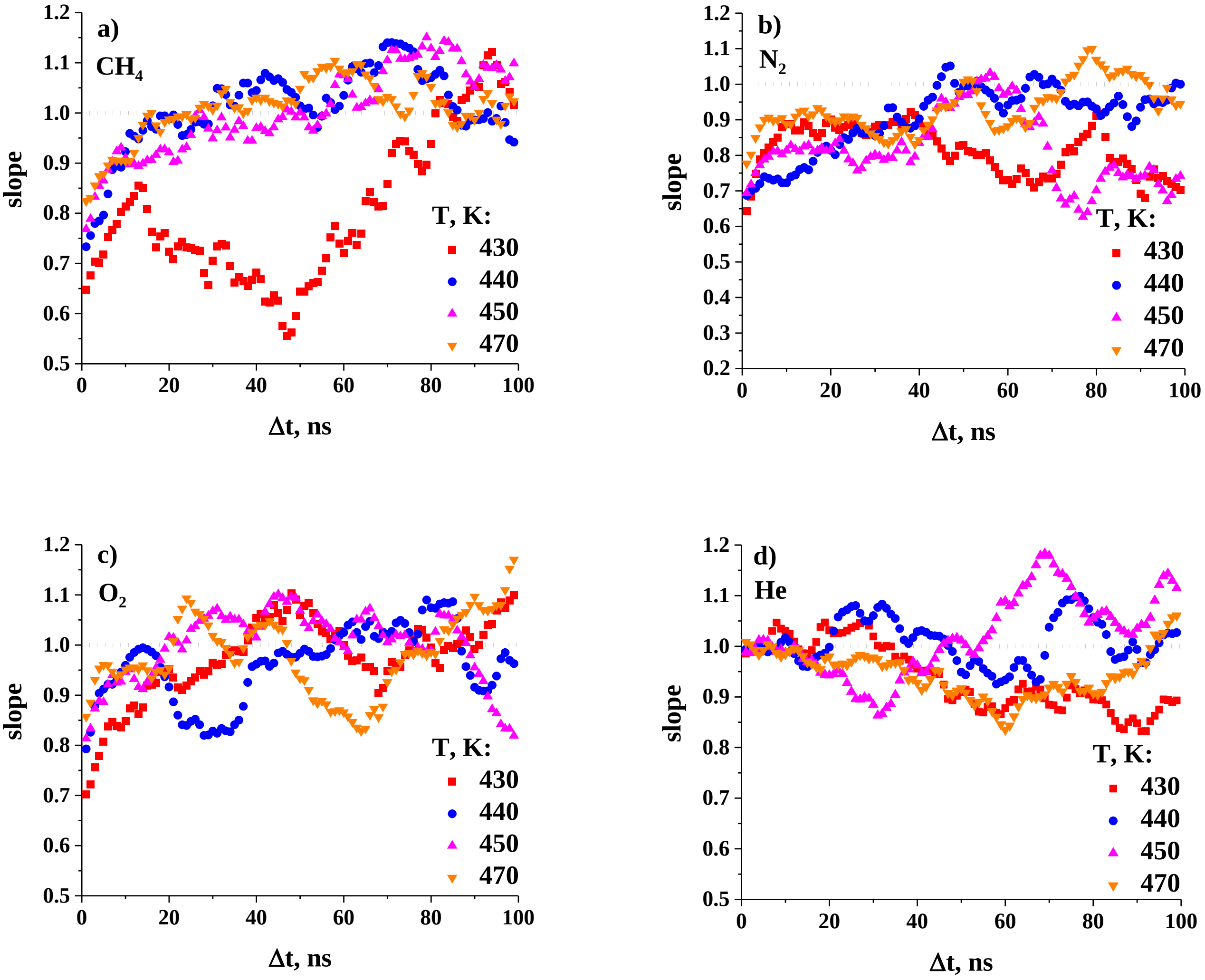

Figure S4. Time-dependence of the slope of the mean squared displacement of a) CH_4, b) O_2, c) N_2, or d) He gas molecules in R-BAPB at different temperatures (the number of gas molecules in the system is equal 10).
